# Intimate partner violence victimization during pregnancy increases risk of postpartum depression among urban adolescent mothers in South Africa

**DOI:** 10.1186/s12978-023-01605-z

**Published:** 2023-05-02

**Authors:** Luwam T. Gebrekristos, Allison K. Groves, Luz McNaughton Reyes, Dhayendre Moodley, Mags Beksinska, Suzanne Maman

**Affiliations:** 1 Department of Epidemiology and Biostatistics, Drexel University Dornsife School of Public Health, 3215 Market Street, Philadelphia, PA 19140 USA; 2 Department of Community Health and Prevention, Drexel University Dornsife School of Public Health, Philadelphia, PA USA; 3 Department of Health Behavior, University of North Carolina at Chapel Hill Gillings School of Global Public Health, Chapel Hill, NC USA; 4 Department of Obstetrics and Gynaecology, University of KwaZulu-Natal Nelson R. Mandela School of Medicine, Durban, South Africa; 5 MatCH Research Unit (MRU), Faculty of Health Sciences, University of the Witwatersrand, Durban, South Africa

**Keywords:** Adolescent mothers, Postpartum depression, Intimate partner violence, Pregnancy, South Africa, Maternal health

## Abstract

**Background:**

It is estimated that 38.8% of mothers develop postpartum depression (PPD) in South Africa. While empirical evidence documents an association between intimate partner violence (IPV) victimization in pregnancy and PPD among adult women, the association has been underexamined among adolescent mothers (< 19 years). The study’s purpose is to examine whether IPV victimization during pregnancy is associated with PPD among adolescent mothers.

**Methods:**

Adolescent mothers (14–19 years) were recruited at a regional hospital’s maternity ward in KwaZulu Natal, South Africa between July 2017-April 2018. Participants completed behavioral assessments at two visits (n = 90): baseline (up to 4 weeks postpartum) and follow-up (6–9 weeks postpartum, when PPD is typically assessed). The WHO modified conflict tactics scale was used to create a binary measure of any physical and/or psychological IPV victimization that occurred during pregnancy. Participants with scores ≥ 13 on the Edinburgh Postpartum Depression Scale (EPDS) were classified as having symptoms of PPD. We used a modified Poisson regression with robust standard errors to assess PPD in association with IPV victimization during pregnancy, controlling for relevant covariates.

**Results:**

Nearly one-half (47%) of adolescent mothers reported symptoms of PPD by 6–9 weeks post-delivery. Further, IPV victimization during pregnancy was highly prevalent (40%). Adolescent mothers who reported IPV victimization during pregnancy had marginally higher risk of PPD at follow-up (RR: 1.50, 95 CI: 0.97–2.31; p = 0.07). The association was strengthened and significant in covariate-adjusted analysis (RR: 1.62, 95 CI: 1.06–2.49; p = 0.03).

**Conclusions:**

Poor mental health was common among adolescent mothers, and IPV victimization during pregnancy was associated with PPD risk among adolescent mothers. Implementing IPV and PPD routine screenings during the perinatal period may aid in identifying adolescent mothers for IPV and PPD interventions and treatment. With the high prevalence of IPV and PPD in this vulnerable population and the potential negative impact on maternal and infant outcomes, interventions to reduce IPV and PPD are needed to improve adolescent mothers’ well-being and their baby’s health.

## Plain language summary

**Background:**

More than one-third of adult mothers experience postpartum depression (PPD) in South Africa and intimate partner violence (IPV) victimization is a strong risk factor of PPD for adult mothers. However, there are no studies on adolescent mothers that look at the link between IPV victimization and PPD. This paper aims to examine whether IPV victimization during pregnancy is associated with PPD among adolescent South African mothers.

**Methods:**

We had 90 adolescent mothers (aged 14–19 years old) complete an initial survey between delivery and 4 weeks postpartum to collect information on IPV during their pregnancy. Participants completed an additional survey between 6 and 9 week postpartum to collect information on the symptoms of PPD.

**Results:**

Nearly one-half (47%) of adolescent mothers reported symptoms of PPD by 6–9 weeks post-delivery. Report of IPV victimization during pregnancy was also very high (40%). Adolescent mothers who experienced IPV victimization during pregnancy were more likely to report symptoms of PPD between 6 and 9 weeks postpartum.

**Conclusions:**

PPD and IPV was very common in our sample, and IPV victimization during pregnancy was linked to PPD among adolescent mothers. Having routine screenings during pregnancy and postpartum period can identify adolescent mothers IPV and PPD interventions and treatment. With the high reports of IPV and PPD in this sample and the potential negative impact on maternal and infant outcomes, interventions to reduce IPV and PPD are needed to improve adolescent mothers’ well-being and their baby’s health.

## Background

The estimated global postpartum depression (PPD) prevalence is 17.2% (95% CI: 16.0–18.5) and higher in low- and middle-income countries [1, 2]. Existing research has linked PPD to numerous negative infant-related outcomes: impaired cognitive or motor development [3–5], low-growth trajectory [3, 6–8], and behavioral inhibition and delayed emotional development [4, 9–11]. Further, PPD negatively impacts maternal social wellbeing (e.g., homelessness, anxiety, substance abuse and suicidal ideation) [12–14] and mother-infant bonding [15, 16].

The prevalence of PPD among adult mothers in South Africa is more than double the global rate (38.8%; 95% CI: 25.7%–53.7%) [1]. Despite the negative infant and maternal consequences of PPD, it is underdiagnosed and routine screening is not a part of standard postpartum care in South Africa [17, 18]. Extant literature documents prior history of depressive symptoms [19–21], unwanted pregnancy [19, 22, 23], being unmarried [19], intimate partner violence (IPV) victimization [22, 24], socioeconomic vulnerabilities [24, 25], and low social support [19–25] as risk factors for PPD in South Africa.

While these studies highlight the prevalence and risk factors of PPD among South African adult mothers, there are gaps in knowledge of the prevalence of PPD and risk factors of PPD for adolescent mothers. One cross-sectional study limited to adolescent mothers (aged 14–19) estimated prevalence of PPD at 8.8% [26]. However, PPD was measured at 1 week postpartum, which is not the standard timing for PPD assessment (4–6 weeks postpartum) [27]. The same study identified IPV victimization, verbal abuse, and physical violence as risk factors of PPD among adolescent mothers [26]. However, with the cross-sectional study design, establishing whether violence was a cause or consequence of PPD is not possible.

Adolescent mothers are largely excluded from studies on postpartum mental health; and yet, the unique circumstances associated with adolescent pregnancy in South Africa may heighten adolescent mothers’ risk of PPD. First, structural factors in post-apartheid South Africa impact the mental health of South Africans, particularly those who are marginalized like black adolescent mothers [28–31]. Legacies of apartheid, like material inequalities, are common among Black adolescent mothers and are risk factors of poor mental health [20, 32, 33]. Second, in two qualitative studies, South African adolescent mothers describe how lack of support and isolation by partners, families and communities during pregnancy led them to experience poor mental health postpartum [34, 35]. At the same time, adolescent mothers are more likely to test positive for HIV during the perinatal period than adult mothers in sub-Saharan Africa [36], which can result in depressive symptoms [37]. Finally, adolescent mothers can experience social stigma which may inhibit engagement in postpartum clinic-based care [38, 39]. Poor engagement in postpartum care may lead to large diagnosis and treatment gaps for adolescent mothers.

Adolescent mothers are usually excluded from studies, yet the unique circumstances,- including the way their intimate relationships are configured – may heighten their risk of PPD. Specifically, several studies demonstrate that adolescent mothers are more likely to have risk factors for PPD compared to adult mothers in that they are more likely to: experience IPV victimization, have relationships with older men (who may be more likely to perpetrate IPV than peer-aged partners) [40], be unmarried [41], and have an unintended pregnancy [41]. These risk factors of PPD are either relationship characteristics or can negatively impact power in their relationships, which highlights the importance of examining relationship characteristics in understanding PPD among adolescent mothers. For example, in population-based studies across several countries women who had an unintended pregnancy were more likely to experience IPV victimization than those who had an intended pregnancy [42].

Some adolescent mothers fare better on risk factors of PPD than other adolescent mothers, which emphasizes the need to conduct research in this population. In South Africa, where the adolescent birth rate is 1.5 times more the global rate [43], risk factors for PPD like unintended pregnancies and being unmarried are extremely common among adolescent mothers [41, 44]; however, there is evidence of variability in IPV victimization. Despite this, no longitudinal studies have examined the link between IPV victimization during pregnancy and symptoms of PPD among adolescent mothers. Understanding the burden of PPD and the link between IPV victimization and symptoms of PPD among this population is needed to identify which adolescent mothers are most at-risk. Thus, the objective of this study is to test the hypothesis that experiencing IPV victimization during pregnancy increases the likelihood of reporting symptoms of PPD among adolescent mothers. This study addresses the current gaps in the literature by leveraging longitudinal data to estimate the prevalence of PPD at 6–9 postpartum and examine the association between IPV during pregnancy and PPD among adolescent mothers in South Africa.

## Methods

### Study design

Data for the analyses comes from a pilot study, Mentoring Adolescent Mothers at School (MAMAS). The MAMAS study was designed to support adolescent mothers return to school following delivery [45]. Screening for eligibility occurred between July 2017 and April 2018 at the regional hospital’s maternity ward in KwaZulu Natal, South Africa. Mothers were eligible to participate in MAMAS if they had attended school in the past year, were between 14 and 19 years old, neither they nor their baby had any major health issues and had planned to live in the township for 6 months (n = 119).

Eligible and interested participants (and parents/guardians of minors) provided informed consent and/or assent prior to initiation of study activities [46]. All enrolled participants completed a STI testing and behavioral survey at their baseline visit (1 day to 4 weeks postpartum) and follow-up visit (between 6–9 weeks postpartum). Participants in the intervention arm were invited to participate in a pilot intervention which consisted of up to 10 group mentoring sessions facilitated by a mentor mother (a young adult mother who had been pregnant as an adolescent and completed high school after delivery). Sessions were delivered from 6 weeks to 6 months postpartum. All study activities were approved by the institutional ethic boards at Drexel University (1612005048) and University of KwaZulu Natal (BFC023/17).

### Measures

The dependent variable in this study was whether the participant reported symptoms of PPD in the past seven days. Participants completed the 10-item Edinburgh Postnatal Depression Scale (EPDS) between 6 and 9 weeks postpartum to identify those with depressive symptoms in the past week [47]. EPDS items were scored on a four-point Likert scale with higher scores indicating concerning symptoms. Scores for the 10 items were summed to generate a final score between 0 and 30 (α = 0.75). A cutoff score of ≥ 13 has been used to indicate depressive symptoms; [47, 48] participants with scores ≥ 13 were coded as having symptoms of PPD. Those with scores < 13 were coded as having no symptoms of PPD.

The independent variable, IPV victimization during pregnancy, was measured retrospectively at baseline (no more than 4 weeks postpartum) using 10 items from the WHO modified conflict tactics scale on physical and psychological violence [49]. Participants reported the frequency at which they experienced each of the 10 acts of violence (e.g., during your pregnancy, has a romantic or sexual partner ever push or shoved you). Adolescent mothers who reported at least 1 act of physical or psychological violence were coded as experiencing IPV victimization during pregnancy. Those who did not report any acts of physical or psychological violence were coded as no IPV victimization during pregnancy.

We controlled for baseline covariates that we hypothesize would be associated with the independent and dependent variables: age (in years) [41, 50–52], socioeconomic status (SES) [25, 53], HIV status [54–56], STI diagnosis [57, 58], perceived social support [20, 25, 26, 59], number of children [60, 61], and symptoms of PPD [20, 26]. We created an index for baseline SES [62, 63], composed of 18 items of working household facilities (e.g., hot running water, electricity, refrigerator). A principal component analysis of the 18 items loaded into a single principal component. Scores lower than the 40th percentile were coded as low SES, scores between 40 and 80th percentile were coded as middle SES, and scores above the 80th percentile were coded as high SES. In South Africa, HIV testing occurs during antenatal care visits and delivery [64]. Participants’ HIV status was determined by reviewal of maternity health records. During the baseline visit, participants completed STI testing for 3 pathogens: Neiserria gonorrhea (Gonorrhea), Chlamydia trachomatis (Chlamydia), and Trichomonas vaginalis (Trichomonas). Participants who tested positive for at least 1 pathogen were coded as having an STI diagnosis. Participants who tested negative on all 3 pathogens were coded as not having an STI diagnosis. Perceived social support was measured using 12-items to assess support from family, friends, and partner (e.g., My family really tries to help me; I can talk about my problems with my friends; I have a romantic partner who is a real source of comfort to me). Items were scored on a 4-point Likert scale with higher scores indicating higher social support. Number of children at the time of their delivery was determined by reviewing maternity health records: 1 child versus 2 children (none of the participants had more than 2 children). Symptoms of PPD within 4 weeks postpartum, like the dependent variable, was measured using EPDS (scores ≥ 13 versus scores < 13, α = 0.82). Finally, we also controlled for time between baseline and follow-up visits (in weeks) and intervention attendance (attended at least one session versus attended zero sessions).

### Data Analysis

We excluded participants who did not complete the follow-up visit (n = 25). We further excluded participants who were missing covariates or the outcome (n = 4), yielding a total sample size of 90 participants. Neither IPV during pregnancy nor baseline symptoms of PPD were associated with study dropout. For our analyses, we used bivariate and multivariable modified Poisson regression with robust standard errors (generates risk ratios) to assess whether IPV victimization during pregnancy is associated PPD, controlling for covariates (α = 0.05) [65, 66]. All analyses were conducted using SAS Version 9.4. [67]

## Results

Nearly half of adolescent mothers reported symptoms of PPD between 6 and 9 weeks postpartum (Table 1; 46.7%) and approximately two-thirds had symptoms of PPD at 4 weeks postpartum or less (65.6%). More than one-third of adolescent mothers reported experiencing IPV during pregnancy (40.0%), with more than one-third reporting any psychological violence (36.7%) and nearly one in five adolescent mothers reporting physical violence (16.7%). Nearly one quarter of participants were HIV-positive (23.3%) and 17.8% of participants had an STI diagnosis at baseline.

**Table 1:** Characteristics of study participants (N = 90)

	All participants N = 90 n(%) or mean (sd)
Dependent variable	
Symptoms of PPD at follow-up	42 (46.7%)
Independent variable	
IPV during pregnancy	36 (40.0%)
Baseline covariates	
Age (in years)	17.5 (1.4)
SES	
Low	37 (41.1%)
Middle	35 (38.9%)
High	18 (20.0%)
Positive HIV status	21 (23.3%)
STI diagnosis	16 (17.8%)
Perceived Social Support Score	3.2 (0.5)
Two children at time of delivery	10 (11.1%)
Symptoms of PPD within 4 weeks postpartum	59 (65.6%)
Attended at least 1 intervention session	39 (43.3%)
Timing between baseline and follow-up (in weeks)	5.4 (1.4)

Table 2 illustrates bivariate associations between IPV, baseline covariates and PPD (Model 1). IPV during pregnancy was marginally associated with symptoms of PPD at follow-up (risk ratio (RR): 1.50; 95% CI: 0.97, 2.31). The risk of PPD symptoms at follow-up was more than double among adolescent mothers who reported symptoms of PPD within 4 weeks postpartum than those who did not report symptoms of PPD within 4 weeks postpartum (RR: 2.23; 95% CI: 1.18, 4.22). No other baseline covariates were associated with PPD at follow up.

**Table 2:** Bivariate and Multivariable Associations between IPV during pregnancy, baseline covariates and symptoms of PPD at follow-up

	Model 1^a^		Model 2^b^	
	RR (95% CI)	p-value	RR (95% CI)	p-value
IPV during pregnancy	1.50 (0.97, 2.31)	0.07	1.62 (1.06, 2.49)	0.03
Age (in years)	1.06 (0.89, 1.27)	0.51	0.99 (0.82, 1.20)	0.95
SES				
Low	Ref.		Ref.	
Middle	1.12 (0.68, 1.86)	0.65	1.04 (0.66, 1.64)	0.86
High	1.16 (0.64, 2.09)	0.63	1.13 (0.60, 2.14)	0.71
HIV-positive	1.31 (0.83, 2.08)	0.24	1.33 (0.82, 2.18)	0.25
STI diagnosis	0.77 (0.39, 1.51)	0.45	1.00 (0.54, 1.85)	0.99
Perceived Social Support Score	0.92 (0.62, 1.37)	0.69	0.93 (0.60, 1.44)	0.75
Two children at time of delivery	1.08 (0.56, 2.10)	0.82	0.96 (0.50, 1.83)	0.9
Symptoms of PPD within 4 weeks postpartum	2.23 (1.18, 4.22)	0.01	2.49 (1.31, 4.72)	0.01
Attended at least 1 intervention session	0.89 (0.56, 1.40)	0.61	0.82 (0.49, 1.38)	0.46
Timing between baseline and follow-up (in weeks)	0.98 (0.82, 1.15)	0.77	0.91 (0.78, 1.07)	0.27

*IPV* intimate partner violence

*PPD* postpartum depression

*RR* risk ratios

^a^Model 1: Bivariate associations

^b^Model 2: Multivariable associations

Table 2 also shows multivariable associations (Model 2). The association between IPV during pregnancy and symptoms of PPD at follow-up was strengthened when controlling for baseline covariates. When accounting for baseline covariates, adolescent mothers who experienced IPV during pregnancy were 1.62 times as likely to report symptoms of PPD at follow-up than those who did not experience IPV during pregnancy (95% CI: 1.06, 2.49). Further, adolescent mothers who had symptoms of PPD within 4 weeks postpartum were 2.49 times as likely to report symptoms of PPD at follow-up compared to those who did not have symptoms of PPD within 4 weeks postpartum (95% CI: 1.31, 4.72) when controlling for other variables.

## Discussion

Despite the high adolescent birth rate and unique context of adolescent pregnancy, the association between IPV and PPD has been understudied among adolescent mothers in South Africa. The purpose of the study was to measure the prevalence of PPD symptoms and examine the link between IPV during pregnancy and symptoms of PPD among adolescent mothers in South Africa. In this study, almost half of adolescent reported symptoms of PPD between 6 and 9 weeks postpartum. Further, experiencing IPV during pregnancy was associated with an increased likelihood of reporting symptoms of PPD.

Studies on PPD among adolescent mothers in sub-Saharan Africa do not measure PPD consistently. Specifically, in two studies, time since delivery varied among study participants and another study measured PPD at 1 week postpartum [26, 68, 69]. Given the variability in PPD assessment within and across studies, it is difficult to compare our study findings to existing studies with adolescent mothers. Nonetheless, our prevalence estimate was higher than depression prevalence estimates among South African adolescent girls (7.3–35.9%) [70–73] and was on the higher end of PPD prevalence estimates among adult mothers (16.7–49.3%). [17, 20–22, 74]

Consistent with our hypothesis, experiencing IPV victimization during pregnancy was associated with the increased likelihood of reporting symptoms of PPD between 6–9 weeks in covariate-adjusted analysis. However, bivariate analysis for IPV victimization was only marginally significant. The reason for statistically significant association in the covariate-adjusted analysis is unclear but may be attributed to negative confounding. Nonetheless, our findings highlight the pervasiveness of IPV victimization among this population and the need for adolescent-friendly IPV screenings and interventions in maternal health care.

In addition to experiencing IPV victimization during pregnancy, reporting symptoms of PPD within 4 weeks postpartum was also associated with increased likelihood of reporting symptoms of PPD between 6 and 9 weeks. Studies in sub-Saharan Africa on adult and adolescent mothers’ mental health have identified history of poor mental health as a predictor of poor postpartum mental health [19, 75, 76]. Further, nearly two-thirds of our sample reported symptoms of PPD within 4 weeks postpartum. Though depressive symptoms in the first month postpartum may indicate baby blues instead of PPD, it was our strongest predictor of PPD between 6 and 9 weeks postpartum. Our findings—along with the broader empirical literature—suggest that we might consider repeated screenings for poor maternal mental health more throughout the perinatal period. Recognizing that there is limited research on postpartum mental health among adolescent mothers, there are several opportunities for future research. While the prevalence of IPV victimization is high, IPV experiences and relationship dynamics are not monolithic in this population. Thus, future research is needed to understand which adolescent mothers are most at-risk for PPD by examining other salient relationship factors beyond IPV, like partner support. Further research is also needed to identify protective factors that mitigate adolescents’ risk of violence during pregnancy and poor mental health postpartum. Finally, given the South African context, further research on structural factors (e.g., income inequality) impact on PPD among adolescent mothers is needed.

In addition, there are several policy and intervention implications. Specifically, clinical providers should implement screenings for IPV during antenatal care visits and screenings for symptoms of PPD during postpartum visits. Despite WHO recommendations [77, 78], routine screenings for PPD and IPV are not included in guidelines for standard of postpartum care in South Africa [18]. Recognizing that optimally-timed screenings is a starting point for additional care rather than an end point, screening without referrals for psychosocial interventions or treatment is not recommended [77]. Acknowledging that adolescent mothers may face stigma by healthcare workers, a barrier to engaging in care [38, 39], providing adolescent-friendly maternal care may increase access to mental health care. Given the lack of interventions that address the specific needs of adolescent mothers, further attention is needed to develop IPV and PPD interventions for adolescent mothers. With the detrimental impact of PPD on both maternal and infant outcomes, it is vital to include adolescent mothers in postpartum mental health research to minimize the burden of IPV victimization and PPD in this vulnerable population.

Our study has several limitations. First, our small sample size precludes our ability to examine physical and psychological IPV victimization separately. It is possible that physical and psychological IPV victimization impact PPD differently. Relatedly, we could not examine sexual IPV victimization. Second, our independent variable, symptoms of PPD, is self-reported not a clinical diagnosis. Although the self-report measure does not replace a clinical diagnosis, studies have shown that EPDS is a strong predictor for clinical diagnosis of PPD [79, 80]. Finally, adolescent mothers were ineligible to participate in the parent study if they reported poor maternal or birth outcomes. Therefore, our findings may not be generalizable to adolescent mothers with poor maternal or birth outcomes. Considering that poor birth outcomes are associated with symptoms of PPD in adult mothers [25], we expect the prevalence of PPD symptoms to be higher in the excluded population.

Nonetheless, this study has many strengths. Our study highlights an overlooked population in postpartum mental health research—adolescent mothers. Further, this study is the first to examine the relationship between IPV during pregnancy and PPD among adolescent mothers in South Africa. Further, we used longitudinal design. Finally, we used a validated and WHO-recommended screener, EPDS, to measure our outcome.

## Conclusions

The burden of PPD and adolescent birth rate is elevated in South Africa. However, adolescent mothers are excluded from postpartum mental health research. Given the unique and difficult circumstances pregnant adolescent girls and adolescent mothers face and the high burden of teen pregnancy in South Africa [81, 82], it is crucial to examine adolescent postpartum mental health. This study broadens our understanding by estimating prevalence of PPD and the impact of IPV victimization on PPD. Our findings show that nearly half of adolescent mothers reported symptoms of PPD and that IPV victimization during pregnancy is associated with increased likelihood of reporting symptoms of PPD. Given the high prevalence of IPV and PPD in this vulnerable population and the negative impact on maternal and infant outcomes, interventions to reduce IPV and PPD are needed to improve adolescent mothers’ well-being and their baby’s health.

## Data Availability

Data used for analyses are available from the corresponding author with reasonable, written request.

## Abbreviations

EPDS: Edinburgh Postnatal Depression Scale
IPV: Intimate partner violence
MAMAS: Mentoring Adolescent Mothers at School
PPD: Postpartum depression
SES: Socioeconomic status
STI: Sexually transmitted infections
